# The perpendicular gyroscope: modal analysis of plate, beam and gyroscope multistructures

**DOI:** 10.1098/rsta.2023.0358

**Published:** 2024-09-23

**Authors:** K. H. Madine, D. J. Colquitt

**Affiliations:** ^1^ Department of Mathematical Sciences, University of Liverpool, Liverpool, UK; ^2^ Department of Materials Engineering, IPHD Program, National Tsing Hua University, Hsinchu City, Taiwan

**Keywords:** gyroscopes, flexural beams and plates, chirality, finite element analysis

## Abstract

This paper presents a study of the perpendicular gyroscope, which is formed of two orthogonal beams, a flexural plate and a gyroscope. Two sets of chiral-torsional boundary conditions are derived to analytically model the dynamic effects of the gyroscope while taking into account the broken symmetries of the system. The perpendicular junction causes the coupling of the compressional, flexural and torsional displacements in the system. This complex behaviour is accounted for with a comprehensive set of kinematic and dynamic junction conditions. Modal analysis demonstrates the fully coupled system and reveals how the spinning gyroscope induces dynamic chiral Chladni patterns in the plate.

This article is part of the theme issue ‘Current developments in elastic and acoustic metamaterials science (Part 2)’.

## 1. Introduction

**Figure 1.**
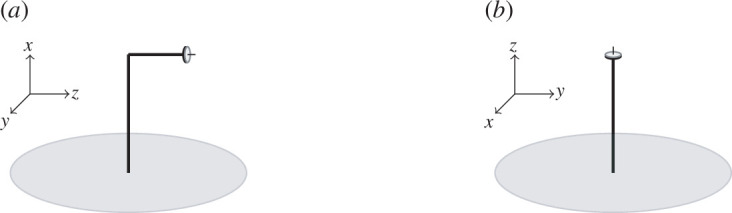
(*a*) The perpendicular gyroscope model, and (*b*) the parallel gyroscope model, both mounted on circular Kirchhoff plates.

Modelling gyroscopic rotations has often proved a complicated endeavour, despite the fact that there are many real-world systems that exhibit gyroscopic motion, such as aeroplanes, wind turbines, motorcycles, navigation devices and propellers for boats and planes. In this work, a combination of analytical and finite element techniques are used to model the *perpendicular gyroscope*, which is formed of two orthogonal beams—with the vertical beam either clamped at the base or mounted on a flexural plate—and a gyroscopic spinner at the end of the horizontal beam, as shown in figure 1*a*. The motivation for the study came from the potential for this work to assist in building future models of gyroscopic systems where the axis of rotation is not parallel to the base, such as for wind turbines. The work in this paper lays the foundation for studying periodic arrays of perpendicular gyroscopes, similar to those found in wind farms around the world. Furthermore, perpendicular gyroscopes provide a mechanism for the implementation of chiral interfaces on arrays which have produced many novel effects not found in traditional metamaterials such as those in [1–3], which are discussed. To the best of our knowledge, this is the first work to incorporate a perpendicular junction between beams when studying combinations of beams, plates and gyroscopes. Moreover, this work demonstrates that the coupling of compressional, flexural and torsional displacements across the perpendicular junction has a significant effect on all aspects of the system, including the eigenmodes, plate displacements and dynamic behaviour of the model.

In recent years, there have been a growing number of studies incorporating gyroscopic spinners in mechanical structures. This has been, at least in part, owing to the ability to dynamically control the chirality of the system. A chiral object is defined as one that cannot be superimposed onto its mirror image [4]. The term ‘passive chirality’ may be used to describe systems that are chiral because of their geometry, such as left and right hands, or left and right shoes. There has been a great deal of interest in passive chirality for photonic metamaterials, employing molecules with geometric chirality to control the propagation of electromagnetic waves, as outlined in the reviews [5,6]. Gyroscopes, however, are associated with ‘active chirality’ because it is their spin that induces chirality. Two indistinguishable gyroscopes that are stationary (not spinning), or are spinning in the same direction are *not* chiral, because they are identical. Conversely, if the gyroscopes are spinning in opposite directions, they are mirror images but cannot be superimposed and so are chiral [4].

The active chirality of gyroscopes was studied in [1], where an infinite flexural plate was topped with a doubly periodic array of beams and gyroscopes. The beams were orientated vertically and each an upright gyroscope placed at the tip, as shown in figure 1*b*. In this paper, to emphasize the difference in the orientation of the spinner with the perpendicular gyroscope, beams with upright gyroscopes at the tips are referred to as *parallel gyroscopes*. Dividing the array of parallel gyroscopes from [1] in half—with the gyroscopes spinning in opposite directions on either side of the divide—produced chiral interfaces and unidirectional modes at the interface in response to applied forcing. Similar highly localized modes on chiral arrays were also studied in [2], where the chiral resonators took the form of two parallel gyroscopes stacked in series on top of flexural plates. The localization of modes along the chiral interfaces was sufficiently strong to bend the waves around corners. Chiral interfaces were also studied in [3,7,8], where triangular and hexagonal lattices of beams had tilted gyroscopes placed at the junctions between neighbouring beams. In particular, the paper [3] demonstrated chiral inter-facial waves that travelled in closed loops, increasing the amplitude of the initial waveform.

The systems of beams and gyroscopes discussed in this work are not to be confused with gyroelastic beams, that is, flexural beams with a continuous distribution of stored angular momentum [9]. The dynamics of gyroelastic beams were studied in [10–12], including comparisons between gyroelastic Euler–Bernoulli beams and gyroelastic Timoshenko beams from [13]. Gyroelastic beams have been used in the proposed design of earthquake protection systems [14] and as a means to help control the flight of spacecraft [15]. While beams with gyroscopes and gyroelastic beams are constitutionally different, there is room for the translation of ideas between the two systems. For example, in [16], the gyro-hinge structure was defined where the pinned end of a flexural beam was connected to a gyroscopic spinner. A periodic array of beams with gyro-hinges was shown to be a good discrete approximation of a gyroelastic beam. Chains of gyropendulums (hanging rods with gyroscopes at the tips) have also been used to approximate continuous gyroscopic ropes, with particular attention paid to the effects of gravity on the system [17].

Both [1] and [2] made use of an important set of boundary conditions that were derived by Nieves, Carta and colleagues in [16,18], called the ‘chiral boundary conditions’. This set of boundary conditions provides forces and moments that account for the presence of a spinner in a parallel gyroscope system while containing all the necessary information relating to its motion—such as its mass, rate of spin and moments of inertia—without having the model the full gyroscope or solve the equations of motion for the spinner. One of the significant differences between parallel and perpendicular gyroscopes is that the former has rotational symmetry about the spin axis, whereas the latter does not. The perpendicular gyroscope’s inherent lack of symmetry results in coupling between flexural, torsional and compressional motion, which, in previous works studying parallel gyroscope systems, were all decoupled. Because of this, the original chiral boundary conditions from [16,18] cannot be immediately applied in this work, so we present a modified set of conditions to account for the torsion. Torsional interactions are often neglected when studying flexural systems insofar as the two are treated independently. However, studying the coupling between flexural and torsional interactions on lattices of beams has shown unusual results, including the ability to control wave propagation on the lattices [19–21].

The structure of the paper is as follows: in §2, we pay close attention to the directions of rotations about the coordinate axes while discussing the equations of motion to account for the broken symmetries of the perpendicular gyroscope system. Careful consideration is given to the coupling of flexural and torsional rotations, leading to sets of dynamic and kinematic conditions in §3, which describe the junction of two perpendicular beams connected end to end. A modified set of chiral-torsional boundary conditions (CTBCs) are provided in §4 to model the presence of the gyroscope. In §5, a new set of boundary conditions are derived which describe the forces and moments (flexural, torsional and compressional) applied to the tip of a beam by the presence of an entire second perpendicular beam and gyroscope combination. These boundary conditions are named the *extended chiral-torsional boundary conditions* (Ex-CTBCs) and allow for the entire perpendicular gyroscope to be modelled using a single beam. In §6, modal analysis is performed to study how the behaviour of the system changes with the rate of spin of the gyroscope for different lengths of the horizontal beam. Investigating the displacements of the plate reveals dynamic chiral Chladni patterns that depend on the rate of spin of the gyroscope. Comparisons are also made between the perpendicular and parallel gyroscope systems. Finally, concluding remarks are presented in §7.

## 2. Equations of motion

The perpendicular gyroscope is formed using two massless Euler–Bernoulli beams; one of the beams is orientated vertically parallel to the x -axis, this beam is referred to as the X -beam. The tip of the X -beam is connected to the end of a second horizontal beam (the Z -beam) such that the angle between the two beams at the junction point is always π/2 . A gyroscopic spinner is fixed to the tip of the Z -beam such that the axis of the gyroscope is always parallel to the end of the Z -beam. The gyroscope can rotate freely, such that the angle of spin ψ does not couple to the angle of torsion $\tau_{z}$ in the beam. We neglect the effects of gravity on the system throughout.

**Figure 2.**
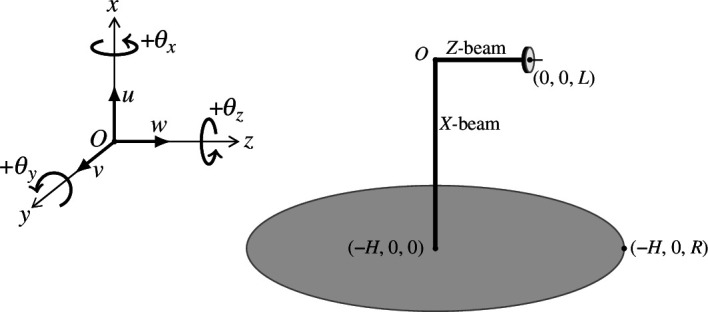
The perpendicular gyroscope on a flexural plate.

We consider two circumstances for the opposite end for the X -beam, either the perpendicular gyroscope is mounted on a Kirchhoff plate, or the X -beam is clamped at the base. We begin by introducing the model with the Kirchhoff plate, then in later sections, set the displacements of the plate to zero, in which case the plate acts as a clamp. The Kirchhoff plate lies in the yz -plane so that the normal of the plate is parallel the x -axis in the undeformed state. The X -beam is cylindrical with cross-sectional radius $a_{x}$ and the end of the beam sits inside an inclusion at the centre of the plate, which also has radius $a_{x}$ . A diagram of a circular Kirchhoff plate with the perpendicular gyroscope at the centre is given in figure 2 with the corresponding coordinate axes. Henceforth, we will use ‘base’ to refer to the connection between the X -beam and plate and ‘junction’ will be exclusively used to refer to point where the X - and Z -beams connect. In later sections we investigate the effect of using a square Kirchhoff plate; however, until stated otherwise, the plate at the base of the X -beam is circular, and the plate is clamped at the boundary. In the following equations, the calligraphic parameters correspond to the X -beam, whereas the standard math typeset parameters refer to the parameters for the Z -beam or the general parameters of the system, plate and gyroscope. It is emphasized that coefficients with tildes always correspond to the X -beam and those without tildes correspond to the Z -beam. Since the system requires the use of many material parameters, a summary is provided in table 1 in appendix A for convenience.

The origin O of the Cartesian coordinate system is located at the junction point where the two beams connect. The displacements u , v and w describe translational motion in the x -, y - and z -directions respectively, as shown in figure 2 with the corresponding positive angles of rotation $\theta_{x}$ , $\theta_{y}$ and $\theta_{z}$ . We emphasize the importance of taking into account the directions of the rotation angles and moments about the coordinate axes, as these become crucial to the later work in this paper. We stipulate that anti-clockwise rotations or moments are defined as positive about their respective axes, as shown in figure 2, and clockwise rotations or moments must gain a negative sign. The height of the X -beam is H , and the length of the Z -beam is L ; the circular plate has radius R and thickness h , thus the clamped boundary of the plate is at $y^{2}+z^{2}=R$ in the x=−H plane. The Kirchhoff plate is massless and its translational motion is limited to the x -direction. Therefore in a time-harmonic regime, the equation of motion for the plate is,


(2.1) $$D\Delta^{2}u_{p}=0\;\;\;\;\text{for}\;\;\;\;\left\{\left(y,z\right):a_{x}\leq y^{2}+z^{2}\leq R\right\},$$

where $u_{p}(y,z)$ is the displacement of the plate in the x -direction and $\Delta^{2}$ is the biharmonic operator. The flexural stiffness of the plate $D=E_{p}h^{3}/12(1-\nu_{p}^{2})$ , where $E_{p}$ is the Young’s modulus and $\nu_{p}$ is Poisson’s ratio of the plate.

The beams experience a combination of flexural, compressional and torsional deformations; we investigate the time-harmonic motion, and omit the factors of exp(iωt) for brevity. For the Z -beam, $u_{z}$ and $v_{z}$ are translational flexural displacements; $w_{z}$ describes the compression along the z -axis and $\tau_{z}$ describes torsional rotation about the z -axis. For the X -beam, ${\tilde{u}}_{x}$ describes the compression along the x -axis, ${\tilde{v}}_{x}$ and ${\tilde{w}}_{x}$ are translational flexural displacements and ${\tilde{\tau }}_{x}$ describes the torsional rotations about the x -axis. In the time-harmonic regime, the flexural displacements of the thin massless beams are governed by,


(2.2) $$EI_{x}\frac{\partial^{4}u_{z}}{\partial z^{4}}=0,\;\;\;\;EI_{y}\frac{\partial^{4}v_{z}}{\partial z^{4}}=0,\;\;\;\;{EI}_{y}\frac{\partial^{4}{\overset{˜}{v}}_{x}}{\partial x^{4}}=0,\;\;\;\;\text{and}\;\;\;\;{EI}_{z}\frac{\partial^{4}{\overset{˜}{w}}_{x}}{\partial x^{4}}=0,$$

where EI and EI are the flexural rigidities for the Z - and X -beams, respectively. The subscripts refer to the direction the second moments of area I and I are measured with respect to. The compressional and torsional displacements satisfy


(2.3) $$EA_{z}\frac{\partial^{2}w_{z}}{\partial z^{2}}=0,\;\;\;\;{EA}_{x}\frac{\partial^{2}{\overset{˜}{u}}_{x}}{\partial x^{2}}=0\;\;\;\;\text{and}\;\;\;\;JG\frac{\partial^{2}\tau_{z}}{\partial z^{2}}=0,\;\;\;\;JG\frac{\partial^{2}{\overset{˜}{\tau }}_{x}}{\partial x^{2}}=0;$$

where EA and EA are the axial stiffnesses, and JG , JG are the torsional stiffnesses of each beam. Furthermore, because the beams are cylindrical, the second moments of area torsion constants are related to the beam radii as follows, $I_{x}=I_{y}=\pi a_{z}^{4}/4$ and $J=\pi a_{z}^{4}/2$ , $I_{y}=I_{z}=\pi a_{x}^{4}/4$ and $J=\pi a_{x}^{4}/2$ [22]. Since equations (2.2) and (2.3) are all homogeneous differential equations, the displacements in the Z -beam can be expressed as the following set of polynomials,


(2.4) $$u_{z}\left(z\right)=u_{3}z^{3}+u_{2}z^{2}+u_{1}z+u_{0},$$

(2.5) $$v_{z}\left(z\right)=v_{3}z^{3}+v_{2}z^{2}+v_{1}z+v_{0},$$

(2.6) $$w_{z}\left(z\right)=w_{1}z+w_{0},$$

(2.7) $$\tau_{z}(z)=\tau_{1}z+\tau_{0}.$$

The displacements of the X -beam are expressed in a similar manner but first we impose the boundary conditions at the base. The translational movement of the X -beam at the base is limited to the motion of the plate at the connection point which is denoted $U_{p}$ . The tilting of the plate about the y - and z -axes has the associated flexural angles $\partial_{z}u_{p}=\varphi_{y}$ and $\partial_{y}u_{p}=\varphi_{z}$ respectively. There is no twisting moment between the beam and the plate about the x -axis. However, the flexural angles $\varphi_{y}$ and $\varphi_{z}$ couple with the flexural angles at the base of the X -beam. Therefore, the kinematic conditions describing the displacements at the base of the X -beam are


(2.8) $$\begin{array}{c} {\tilde{u}}_{x}(-H)=U_{p}\\ {\tilde{v}}_{x}(-H)=0 \end{array}\;\;\;\;\begin{array}{c} {\tilde{w}}_{x}(-H)=0\\ {\tilde{\tau }}_{x}(-H)=0 \end{array}\;\;\;\;\begin{array}{c} \partial_{x}{\tilde{v}}_{x}(-H)=\varphi_{z}\\ \partial_{x}{\tilde{w}}_{x}(-H)=\varphi_{y}. \end{array}$$

With these boundary conditions, the displacements of the X -beam can then be expressed


(2.9) $${\overset{˜}{u}}_{x}\left(x\right)={\overset{˜}{u}}_{1}\left(x+H\right)+U_{p},$$

(2.10) $${\overset{˜}{v}}_{x}\left(x\right)={\overset{˜}{v}}_{3}{\left(x+H\right)}^{3}+{\overset{˜}{v}}_{2}{\left(x+H\right)}^{2}+\varphi_{z}\left(x+H\right),$$

(2.11) $${\overset{˜}{w}}_{x}\left(x\right)={\overset{˜}{w}}_{3}{\left(x+H\right)}^{3}+{\overset{˜}{w}}_{2}{\left(x+H\right)}^{2}+\varphi_{y}\left(x+H\right),$$

(2.12) $${\tilde{\tau }}_{x}(x)={\tilde{\tau }}_{1}(x+H).$$

Until this point, the equations of motion for the Z - and X -beams are written with full derivatives to highlight the fact that the derivatives are taken with respect to z or x for each beam, respectively. For the following work, it is convenient to introduce prime notation to indicate spatial derivatives. The subscript, which denotes the beam the displacement corresponds to, also indicates which variable is used for the derivative, such as in the following examples,


$$-{EI}_{y}{\frac{\partial^{3}{\overset{˜}{v}}_{x}}{\partial x^{3}}|}_{x=0}=-{EI}_{y}{\overset{˜}{v}}_{x}^{\prime \prime \prime }\left(0\right)\;\;\;\;\text{and}\;\;\;\;{\frac{\partial v_{z}}{\partial z}|}_{z=L}=v_{z}^{\prime }\left(L\right).$$

Considering again the connection of the beam and plate, with $a_{x}\ll R$ , the forces and moments applied to the base of the X -beam from the plate at (−H,0,0) are expressed as,


(2.13) $$-{EI}_{y}{\overset{˜}{v}}_{x}^{\prime \prime }\left(-H\right)=\frac{4\pi D}{\text{ln}\left(a_{x}/R\right)}\varphi_{z},$$

(2.14) $${EI}_{z}{\overset{˜}{w}}_{x}^{\prime \prime }\left(-H\right)=-\frac{4\pi D}{\text{ln}\left(a_{x}/R\right)}\varphi_{y},$$

(2.15) $${EA}_{x}{\overset{˜}{u}}_{x}^{\prime }\left(-H\right)=\frac{16\pi D}{R^{2}}U_{p}.$$

These equations are referred to as the logarithmic boundary conditions. They were first derived in [1] for beams parallel to the z -axis and plates in the xy -plane, and so have been rotated and translated to be suitable for the perpendicular gyroscope system. In [1], the original logarithmic boundary conditions were derived from the dynamic Green’s function of heavy plates and used the static limit to arrive at the result. In appendix B of this paper, an alternative—and objectively simpler—derivation of the logarithmic boundary conditions is provided for massless beams and plates by making use of solutions to the biharmonic equation.

## 3. Coupling conditions at the junction between two perpendicular beams

It is important to note that the flexural and torsional displacements couple at the junction between the two beams. Similarly, different classes of forces and moments also couple between the beams at the junction point, these relations are discussed below. Firstly, we consider displacements and rotations at the junction between the X -beam and the Z -beam. The translational displacements are transferred between the two beams in a straightforward manner, with translational displacements in the Z -beam equal to the translational displacements of the X -beam for the corresponding x -, y - and z -directions. Flexural and torsional rotations in beams are most often treated as independent; however, properly accounting for the coupling of rotations between beams has been shown to have significant effects on systems of perpendicular beams [20,21].

For the perpendicular gyroscope, rotations about the z -axis at the junction cause a flexural deformation of the X -beam in the v direction, of magnitude $\theta_{z}$ . The flexural deformation of the X -beam causes rotation of the Z -beam about the z -axis. This angle when considered alone is not a torsional angle about the z -axis for the Z -beam, as this deformation will cause a rotation of the whole Z -beam about the z -axis by $\theta_{z}$ . However, at the opposite end of the Z -beam, the mass of the gyroscope has an associated moment of inertia about the z -axis which resists the twisting motion and so the Z -beam does experience torsional deformation; hence the angles ${\tilde{v}}_{x}^{\prime }(0)$ and $\tau_{z}(0)$ are coupled.

Considering rotations about the y -axis at the junction, a rotation $\theta_{y}$ causes the Z -beam to experience flexural deformation in the u -direction. Since the junction point of the beams is such that the angle between the two beams is always π/2 , the magnitude of the $\theta_{y}$ rotation is equal to the magnitude of flexural rotation experienced by the X -beam away from the negative x -axis in the w direction. This rotation couples the angles $u_{z}^{\prime }(0)$ and ${\tilde{w}}_{x}^{\prime }(0)$ .

For rotations about the x -axis, a ${\tilde{\tau }}_{x}(0)$ torsional deformation of the X -beam, $\theta_{x}$ causes the Z -beam to rotate in the yz -plane about the x -axis at the junction point. Similar to above, this angle alone is purely torsional and acts to rotate the entire Z -beam about the x -axis. However, the translational inertia associated with the mass of the gyroscope induces resistive forces and moments in the Z -beam, and so the Z -beam does experience flexural deformation in the y -direction. Thus the flexural rotation angle $v_{z}^{\prime }(0)$ is coupled with the torsional angle ${\tilde{\tau }}_{x}(0)$ .

Taking these coupling conditions into account leads to the following six kinematic conditions at the junction point,


(3.1) $$\begin{array}{c} {\tilde{u}}_{x}(0)=u_{z}(0)\\ {\tilde{v}}_{x}(0)=v_{z}(0) \end{array}\;\;\;\;\begin{array}{c} {\tilde{w}}_{x}(0)=w_{z}(0)\\ {\tilde{\tau }}_{x}(0)=v_{z}^{\prime }(0) \end{array}\;\;\;\;\begin{array}{c} {\tilde{w}}_{x}^{\prime }(0)=u_{z}^{\prime }(0)\\ {\tilde{v}}_{x}^{\prime }(0)=\tau_{z}(0). \end{array}$$

Second, we consider the forces and moments at the junction. The shear force in the x -direction from the Z -beam couples to the compressional force in the X -beam at the junction, giving (3.2). In the y -direction, the shear forces couple between the two beams, giving the relation (3.3). In the z -direction, compressional force in the Z -beam couples to shear force in the X -beam, leading to (3.4). Therefore, the relations between the forces are


(3.2) $${EA}_{x}{\overset{˜}{u}}_{x}^{\prime }\left(0\right)=-EI_{x}u_{z}^{\prime \prime \prime }\left(0\right),$$

(3.3) $$-{EI}_{y}{\overset{˜}{v}}_{x}^{\prime \prime \prime }\left(0\right)=-EI_{y}v_{z}^{\prime \prime \prime }\left(0\right),$$

(3.4) $$-{EI}_{z}{\overset{˜}{w}}_{x}^{\prime \prime \prime }\left(0\right)=EA_{z}w_{z}^{\prime }\left(0\right).$$

For the moments, flexural moments in the Z -beam about the x -axis couple to torsional moments in the X -beam, leading to (3.5). In both the X - and Z -beams, the moments about the y -axis are flexural bending moments, which gives the relation (3.6). Torsional moments in the Z -beam couple to flexural moments in the X -beam about the z -axis, giving (3.7). All together,


(3.5) $$JG{\overset{˜}{\tau }}_{x}^{\prime }\left(0\right)=EI_{y}v_{z}^{\prime \prime }\left(0\right),$$

(3.6) $$-{EI}_{z}{\overset{˜}{w}}_{x}^{\prime \prime }\left(0\right)=-EI_{x}u_{z}^{\prime \prime }\left(0\right),$$

(3.7) $${EI}_{y}{\overset{˜}{v}}_{x}^{\prime \prime }\left(0\right)=JG\tau_{z}^{\prime }\left(0\right).$$

From the combination of the kinematic conditions (3.1) and balancing the forces and moments above, we have a comprehensive map that describes how displacements, rotations, forces and moments are translated and coupled across the connection between two perpendicular beams connected end to end. This fully accounts for the coupling between torsional and flexural rotations in a system that does not have rotational symmetry, and allows for displacements of the base of the system, which allows for the connection to the plate or a different foundation. We have not yet taken into account the presence of a gyroscope, so in the absence of a spinner, any desired mass, resonator or forces and moments could be attached or applied to the free end of the Z -beam and the above (3.1)–(3.7) would form a standalone model of a perpendicular connection between two flexural beams.

In the following sections, the relationship between the coefficients of the polynomials given by the kinematic conditions (3.1)–(3.7) are used to solve the eigenvalue problem by eliminating sets of variables. Explicit equations for the relationships between the coefficients are given in appendix C but are omitted here for brevity.

## 4. The chiral-torsional boundary conditions

As discussed in §1, the original five chiral boundary conditions of [18] were derived to simulate the presence of a gyroscope at the tip of a single beam in a parallel gyroscope system, see figure 1. Parallel gyroscope systems have rotational symmetry about the axis of the beam; this means that the moments applied by the gyroscope to the tip of the upright beam can be induced either clockwise or anti-clockwise about the in plane axes without affecting the eigenmodes. The torsion in the system can also be neglected without consequence. This, however, is *not* the case for the perpendicular gyroscope, where we must take into account the fact that the Z -beam breaks the rotational symmetry of the system, and so determining the direction of the rotations and applied moments is paramount. In this section, we present set of six modified boundary conditions to describe the effects of a gyroscope placed at the free end of the Z -beam, these are referred to as the *chiral-torsional boundary conditions* (*CTBCs*).

Firstly, considering the shear and compressional forces applied by the gyroscope to the tip of the Z -beam at (0,0,L) , we have the following relations,


(4.1) $$-EI_{x}u_{z}^{\prime \prime \prime }\left(L\right)=m\omega^{2}u_{z}\left(L\right),$$

(4.2) $$-EI_{y}v_{z}^{\prime \prime \prime }\left(L\right)=m\omega^{2}v_{z}\left(L\right),$$

(4.3) $$EA_{z}w_{z}^{\prime }(L)=m\omega^{2}w_{z}(L).$$

The angular frequency of the system is ω , and m is the mass of the gyroscope. Equations (4.1)–(4.3) are unchanged from the original chiral boundary conditions of [18], the difference occurs when considering the rotations at the tip of the Z -beam and the moments applied to the beam by the gyroscope. In particular, the moment about the x -axis must be clockwise to remain consistent with the rest of the system. Furthermore, we note that a positive $v_{z}(L)$ deformation corresponds to a negative angle of rotation which must also be accounted for. Another crucial difference comes from the coupling of flexural and torsional rotations between the perpendicular beams. This requires a new sixth boundary condition—equation (4.6)—to account for the torsional moment associated with the inertia of the gyroscope about the z -axis. We reiterate that the spinning motion of the gyroscope does not induce torsion in the Z -beam, however the rotational inertia arising from the mass of the gyroscope does still affect the torsion in the beam. Considering all of this, balancing the moments at the tip of the Z -beam gives the relations,


(4.4) $$EI_{y}v_{z}^{\prime \prime }\left(L\right)=I_{0}\omega^{2}v_{z}^{\prime }\left(L\right)-\text{i}\omega I_{1}\Omega u_{z}^{\prime }\left(L\right).$$

(4.5) $$-EI_{x}u_{z}^{\prime \prime }\left(L\right)=-I_{0}\omega^{2}u_{z}^{\prime }\left(L\right)-\text{i}\omega I_{1}\Omega v_{z}^{\prime }\left(L\right).$$

(4.6) $$-JG\tau_{z}^{\prime }(L)=-I_{1}\omega^{2}\tau_{z}(L).$$

The constants $I_{0}$ and $I_{1}$ are the moments of inertia of the spinner, with $I_{0}$ being the moment of inertia about the x - and y -axes and $I_{1}$ being the moment of inertia about the z -axis. The constant Ω is the gyricity, which characterizes the rate of spin of the gyroscope when the angle of nutation of the gyroscope is small. The gyricity is precisely defined by the rate of spin ψ , with the rate of precession ϕ of the gyroscope, such that


$$\Omega =\dot{\phi }+\dot{\psi }=\text{const},$$

where the over-dots represent differentiation with respect to time [16,18]. For Ω=0 , the gyroscope is stationary, and so acts like a mass at the end of the Z -beam. Non-zero Ω indicates that the gyroscope is spinning, and the sign of Ω (positive or negative) indicates the direction of spin of the gyroscope (anti-clockwise or clockwise).

Solving the CTBCs as a set of six simultaneous equations allows us to find the eigenfrequencies ω of the perpendicular gyroscope system. We are especially interested in the eigenfrequencies as functions of the gyricity Ω , investigating ω(Ω) allows us to observe how the behaviour of the perpendicular gyroscope changes with the rate of spin. The first step in solving for ω(Ω) is to express (4.1)–(4.6) in terms of the coefficients of the polynomials $u_{z}$ , $v_{z}$ , $w_{z}$ and $\tau_{z}$ ,


(4.7) $$\begin{array}{c} 0=6EI_{x}u_{3}+m\omega^{2}(u_{3}L^{3}+u_{2}L^{2}+u_{1}L+u_{0})\\ 0=6EI_{y}v_{3}+m\omega^{2}(v_{3}L^{3}+v_{2}L^{2}+v_{1}L+v_{0})\\ 0=-EA_{z}w_{1}+m\omega^{2}(w_{1}L+w_{0})\\ 0=6EI_{y}Lv_{3}+2EI_{y}v_{2}-I_{0}\omega^{2}(3v_{3}L^{2}+2v_{2}L+v_{1})+\text{i}\omega I_{1}\Omega (3u_{3}L^{2}+2u_{2}L+u_{1})\\ 0=6EI_{x}Lu_{3}+2EI_{x}u_{2}-I_{0}\omega^{2}(3u_{3}L^{2}+2u_{2}L+u_{1})-\text{i}\omega I_{1}\Omega (3v_{3}L^{2}+2v_{2}L+v_{1})\\ 0=-JG\tau_{1}+I_{1}\omega^{2}(\tau_{1}L+\tau_{0}). \end{array}$$

At this point, the set of 12 coefficients $\{u_{3},u_{2},u_{1},u_{0},v_{3},v_{2},v_{1},v_{0},w_{1},w_{0},\tau_{1},\tau_{0}\},$ are all unknowns. However, returning to §3, the kinematic conditions from (3.1) to (3.7) show that the coefficients of the displacement equations for the Z -beam and the X -beam are intrinsically linked (detailed expressions are given in appendix C). At this point, it is convenient to express the flexural and torsional angles at the junction in terms of general angles at (0,0,0) . It is imperative that the direction of the angle is taken into account because of the broken symmetries of the system, and so we use the relations


(4.8) $$v_{z}^{\prime }(0)=v_{1}=-\theta_{x},\;\;\;\;\;\;\;\;u_{z}^{\prime }(0)=u_{1}=\theta_{y},\;\;\;\;\;\;\;\;\tau_{z}(0)=\tau_{0}=-\theta_{z}.$$

With these relations, we can reduce the system to six unknowns $\left\{u_{0},v_{0},w_{0},\theta_{x},\theta_{y},\theta_{z}\right\}$ , and this in turn allows us to solve the set of simultaneous equations from (4.7). We retain the 0 subscripts on the $u_{0}$ , $v_{0}$ and $w_{0}$ displacements to emphasize that these are the displacements at the junction point (0,0,0) , keeping in mind the relations from (3.1) which state that ${\tilde{u}}_{x}(0)=u_{z}(0)=u_{0}$ , and likewise for the ${\tilde{v}}_{x}(0)$ and ${\tilde{w}}_{x}(0)$ .

Until now, we have modelled a perpendicular gyroscope system mounted on a Kirchhoff plate. Here, we wish to study the eigenmodes of the perpendicular gyroscope system alone, that is, a perpendicular gyroscope which is clamped at the base. Therefore, we set the displacements of the plate $U_{p}=\varphi_{z}=\varphi_{y}=0$ . In terms of the displacements at the junction, the six simultaneous equations for a clamped perpendicular gyroscope system are expressed as follows,


(4.9) $$0=-\frac{{EA}_{x}}{H}u_{0}+mw^{2}\left(-\frac{{EA}_{x}L^{2}}{6EI_{x}H}u_{0}+\frac{{EI}_{z}L^{2}}{2EI_{x}H^{2}}(4\theta_{y}H-6w_{0})+\theta_{y}L+u_{0}\right)\;0=-\frac{6{EI}_{y}}{H^{3}}(\theta_{z}H+2u_{0})+mw^{2}\left(\frac{{EI}_{y}L^{3}}{6EI_{y}H^{3}}(-6\theta_{z}H-12u_{0})-\frac{JGL^{2}}{2EI_{y}H}\theta_{x}-\theta_{x}L+u_{0}\right)\;0=\frac{6{EI}_{z}L}{H^{3}}(-\theta_{z}H-2w_{0})+mw^{2}\left(-\frac{6{EI}_{z}L}{EA_{z}H^{3}}(\theta_{y}H-2w_{0})+w_{0}\right)\;0=\frac{6{EI}_{y}L}{H^{3}}(-\theta_{z}H-2u_{0})-\frac{JG}{H}\theta_{x}-I_{0}w^{2}\left(\frac{3{EI}_{y}L^{2}}{EI_{y}H^{3}}(-\theta_{z}H-2u_{0})-\frac{JGL}{EI_{y}H}\theta_{x}-\theta_{x}\right)\;\;\;\;+I_{1}wi\Omega \left(-\frac{{EA}_{x}L^{2}}{2EI_{x}H}u_{0}+\frac{{EI}_{z}L}{EI_{x}H^{2}}(4\theta_{y}H-6w_{0})+\theta_{y}\right)\;0=-\frac{{EA}_{x}L}{H}u_{0}+\frac{{EI}_{z}}{H^{2}}(4\theta_{y}H-6w_{0})-I_{0}w^{2}\left(-\frac{{EA}_{x}L^{2}}{2EI_{x}H}u_{0}+\frac{{EI}_{z}L}{EI_{x}H^{2}}(4\theta_{y}H-6w_{0})+\theta_{y}\right)\;\;\;\;-iwI_{1}\Omega \left(\frac{3{EI}_{y}L^{2}}{EI_{y}H^{3}}(-\theta_{z}H-2u_{0})-\frac{JGL}{EI_{y}H}\theta_{x}-\theta_{x}\right)\;0=\frac{{EI}_{y}}{H^{2}}(4\theta_{z}H+6u_{0})+I_{1}w^{2}\left(\frac{{EI}_{y}L}{JGH^{2}}(4\theta_{z}H+6u_{0})-\theta_{z}\right).$$

The equations of (4.9) are then written as a matrix of coefficients Q multiplying the vector $r\;={\left[u_{0},v_{0},w_{0},\theta_{x},\theta_{y},\theta_{z}\right]}^{\text{T}}.$ The eigenfrequencies are found through the non-trivial solution of Q r=0 , where 0 is the six-dimensional zero vector, such that detQ=0 . At this point, it is necessary to choose numerical values for each of the material parameters, these are chosen to be in accordance with long thin steel beams, and so $a_{z}=a_{x}=0.02$ m, E=E=70 GPa, and G=G=26 GPa, with H=1 m. We initially set L=0.5 m, although changing the length of the Z -beam will be investigated in later sections of this work. For the gyroscope, m=1 kg, and $I_{0}=4$ kg m ${,}^{2}$ while $I_{1}=2$ kg m ${,}^{2}$ .

**Figure 3.**
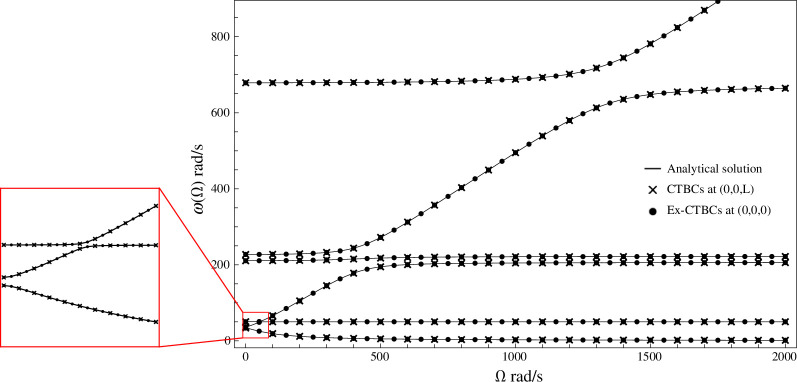
The eigenfrequencies of a perpendicular gyroscope system with a clamped base, as functions of the gyricity. The analytical solution (solid lines) has been overlaid with the results from the two finite element models discussed in §5, demonstrating the agreement of the three models. The crosses indicate the eigenfrequency solutions from the finite element model with the CTBCs applied at the tip of the Z-beam. The dots indicate the eigenfrequency solutions for the finite element model with the Ex-CTBCs applied to the tip of the X-beam. The avoided crossings of the first and second, then second and third eigenfrequency branches are shown inset.

The solutions ω(Ω) of detQ=0 are plotted in figure 3, with some of the eigenfrequency branches showing significant changes as the gyricity increases. For higher values of Ω , the lower five eigenfrequency branches all plateau, indicating a stabilizing effect when the gyroscope is spinning fast. However, the sixth mode—which is associated with gyroscopic motion, as will later be shown—continues to increase proportional to the gyricity without plateauing. The lowest eigenfrequency branch becomes very small as Ω→∞ but continues to show gyroscopic rotations. The eigenfrequency branches are all distinct and do not cross; this includes the first and second eigenfrequencies at Ω =0 rad/s and the second- and third-lowest eigenfrequencies when Ω≈65 rad/s which come very near, however taking a closer look at this region of the graph (inset) shows true avoided crossings. The avoided crossings indicate that all of the modes are linearly dependent and the perpendicular gyroscope system is fully coupled. The distinct eigenfrequencies at Ω =0 rad/s also gives a notable difference to the parallel gyroscope, which has double eigenfrequencies for zero gyricity [18].

## 5. Extended chiral-torsional boundary conditions

Modal analysis of the perpendicular gyroscope for different values of the gyricity is provided in §6. However, in this section, we provide a new set of forces and moments, which are referred to as the extended chiral-torsional bundary conditions (Ex-CTBCs). In a similar manner to how the CTBCs simulate the presence of the gyroscope at the tip of the Z -beam, the Ex-CTBCs applied at the tip of the X -beam simulate the presence of the gyroscope and the Z -beam together.

To derive the Ex-CTBCs, we consider the forces and moments that are applied to the junction by the Z -beam in each direction. The forces in the x -, y - and z -directions respectively are


(5.1) $$\begin{array}{c} -EI_{x}u_{z}^{\prime \prime \prime }(0)=-6EI_{x}u_{3}\\ -EI_{y}v_{z}^{\prime \prime \prime }(0)=-6EI_{x}v_{3}\\ EA_{z}w_{z}^{\prime }(0)=EA_{z}w_{1}. \end{array}$$

The moments about the x -, y - and z -axes are


(5.2) $$\begin{array}{c} EI_{y}v_{z}^{\prime \prime }(0)=2EI_{y}v_{2}\\ -EI_{x}u_{z}^{\prime \prime }(0)=-2EI_{x}u_{2}\\ JG\tau_{z}^{\prime }(0)=JG\tau_{1}. \end{array}$$

We use the CTBCs from (4.7) to determine the contribution of the gyroscope at the junction. Following this, we use the kinematic conditions of (3.1) and the balance of forces from (3.2) to (3.7) to rewrite (5.1) and (5.2) in terms of the displacements at the tip of the X -beam and the displacements of the plate. Balancing the forces gives,


(5.3) $$\begin{array}{c} {EA}_{x}{\overset{˜}{u}}_{x}^{\prime }\left(0\right)=m\omega^{2}\left(-\frac{{EA}_{x}}{6EI_{x}}\frac{L^{3}}{H}\left({\overset{˜}{u}}_{x}\left(0\right)-U_{p}\right)+{\overset{˜}{w}}_{x}^{\prime }\left(0\right)L+{\overset{˜}{u}}_{x}\left(0\right)\;\;\;\;\;+\frac{{EI}_{z}}{2EI_{x}}\frac{L^{2}}{H^{2}}\left(4{\overset{˜}{w}}_{x}^{\prime }\left(0\right)H-6{\overset{˜}{w}}_{x}\left(0\right)+2\varphi_{y}H\right)\right) \end{array}$$

(5.4) $$\begin{array}{c} -{EI}_{y}{\overset{˜}{v}}_{x}^{\prime \prime \prime }\left(0\right)=m\omega^{2}\left(\frac{{EI}_{y}}{6EI_{y}}\frac{L^{3}}{H^{3}}\left(6{\overset{˜}{v}}_{x}^{\prime }\left(0\right)H-12{\overset{˜}{v}}_{x}\left(0\right)+6\varphi_{z}H\right)\;\;\;\;\;\;\;+\frac{JG}{2EI_{y}}\frac{L^{2}}{H}{\overset{˜}{\tau }}_{x}\left(0\right)+{\overset{˜}{\tau }}_{x}\left(0\right)L+{\overset{˜}{v}}_{x}\left(0\right)\right) \end{array}$$

(5.5) $$-{EI}_{z}{\overset{˜}{w}}_{x}^{\prime \prime \prime }\left(0\right)=m\omega^{2}\left(-\frac{6{EI}_{z}}{EA_{z}}\frac{L}{H^{3}}\left({\overset{˜}{w}}_{x}^{\prime }\left(0\right)H-2{\overset{˜}{w}}_{x}\left(0\right)+\varphi_{y}H\right)+{\overset{˜}{w}}_{x}\left(0\right)\right).$$

For the moments,


(5.6) $$\begin{array}{ll} JG{\overset{˜}{\tau }}_{x}^{\prime }\left(0\right) & =I_{0}\omega^{2}\left(\frac{3{EI}_{y}L^{2}}{EI_{y}H^{3}}\left(\varphi_{z}H+{\overset{˜}{v}}_{x}^{\prime }\left(0\right)H-2{\overset{˜}{v}}_{x}\left(0\right)\right)+\frac{JGL}{EI_{y}H}{\overset{˜}{\tau }}_{x}\left(0\right)+{\overset{˜}{\tau }}_{x}\left(0\right)\right)\\ \;\;\;\; & -\text{i}\omega I_{1}\Omega \left({\overset{˜}{w}}_{x}^{\prime }\left(0\right)-\frac{{EA}_{x}L^{2}}{2EI_{x}H}\left({\overset{˜}{u}}_{x}\left(0\right)-U_{p}\right)+\frac{2{EI}_{z}L}{EI_{x}H}\left(2{\overset{˜}{w}}_{x}^{\prime }\left(0\right)-\frac{3}{H}{\overset{˜}{w}}_{x}\left(0\right)+\varphi_{y}\right)\right)\\ & +\frac{6{EI}_{y}L}{H^{3}}\left(-{\overset{˜}{v}}_{x}^{\prime }\left(0\right)H+2{\overset{˜}{v}}_{x}\left(0\right)-\varphi_{z}H\right), \end{array}$$

(5.7) $$\begin{array}{ll} -{EI}_{z}{\overset{˜}{w}}_{x}^{\prime \prime }\left(0\right) & =-\text{i}\omega I_{1}\Omega \left({\overset{˜}{\tau }}_{x}\left(0\right)+\frac{6{EI}_{y}}{2EI_{y}}\frac{L^{2}}{H^{3}}\left(\varphi_{z}H+{\overset{˜}{v}}_{x}^{\prime }\left(0\right)H-2{\overset{˜}{v}}_{x}\left(0\right)\right)+\frac{JG}{EI_{y}}\frac{L}{H}{\overset{˜}{\tau }}_{x}\left(0\right)\right)\\ \;\;\;\;\;\;\;\; & -\frac{{EA}_{x}L}{H}\left({\overset{˜}{u}}_{x}\left(0\right)-U_{p}\right)-I_{0}\omega^{2}\left(\frac{{EI}_{z}}{EI_{x}}\frac{L}{H^{2}}\left(4{\overset{˜}{w}}_{x}^{\prime }\left(0\right)H-6{\overset{˜}{w}}_{x}\left(0\right)+2\varphi_{y}H\right)\;+{\overset{˜}{w}}_{x}^{\prime }\left(0\right)+\frac{{EA}_{x}}{2EI_{x}}\frac{L^{2}}{H}\left(U_{p}-{\overset{˜}{u}}_{x}\left(0\right)\right)\right),\;\;\;\;\;\;\;\;\;\;\;\;\;\; \end{array}$$

(5.8) $${EI}_{y}{\overset{˜}{v}}_{x}^{\prime \prime }\left(0\right)=I_{1}\omega^{2}\left(\frac{{EI}_{y}}{JG}\frac{L}{H^{2}}\left(-4{\overset{˜}{v}}_{x}^{\prime }\left(0\right)H+6{\overset{˜}{v}}_{x}\left(0\right)+2\varphi_{z}H\right)+{\overset{˜}{v}}_{x}^{\prime }\left(0\right)\right).$$

With the Ex-CTBCs (5.1)–(5.8), the entire perpendicular gyroscope system can be modelled using a single upright beam.

In the same manner as was done with (4.8), the displacement angles ${\tilde{\tau }}_{x}(0)$ , ${\tilde{v}}_{x}^{\prime }(0)$ and ${\tilde{w}}_{x}^{\prime }(0)$ can be rewritten in terms of the general angles at the junctions,


(5.9) $${\tilde{\tau }}_{x}(0)=-\theta_{x},\;\;\;\;\;\;\;\;{\tilde{w}}_{x}^{\prime }(0)=\theta_{y},\;\;\;\;\;\;\;\;{\tilde{v}}_{x}^{\prime }(0)=-\theta_{z}.$$

Expressing the displacements at the junction as $u_{0}$ , $v_{0}$ , $w_{0}$ and using the kinematic conditions (3.1) with the displacements of the plate set to zero (such that the plate acts as a clamp) the Ex-CTBCs from (5.3) to (5.8) return precisely the set of simultaneous (4.9). Therefore, solving for the eigenmodes of the perpendicular gyroscope system by applying the Ex-CTBCs to the tip of the X -beam produces the exact graph of ω(Ω) from figure 3.

**Figure 4.**
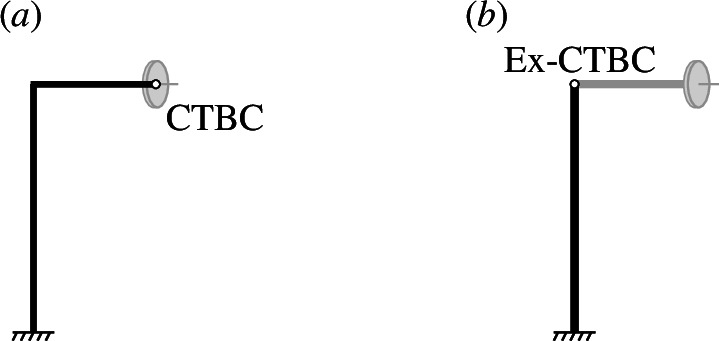
(*a*) Two perpendicular Euler–Bernoulli beams, with the CTBCs applied at the tip of the horizontal beam. (*b*) A single upright Euler–Bernoulli beam with the Ex-CTBCs applied at the tip of the beam to simulate the presence of a horizontal beam with a gyroscopic spinner.

In later sections, finite element models are used to illustrate the dynamic behaviour of the perpendicular gyroscope. These finite element simulations complement the analytical results presented above. In particular, whereas the analytical methods provide physical insight and can capture subtle phenomena that cannot be easily obtained through finite element simulations—such as the avoiding crossings shown in figure 3—finite element models are particularly useful for demonstrating the overall mode shapes and qualitative behaviour of the perpendicular gyroscope. Given the importance of ensuring the finite element models are comprehensive, accurate and fully encapsulate the perpendicular gyroscope system, we demonstrate the application of both the CTBCs and Ex-CTBCs with finite element simulations. Using COMSOL Multiphysics^®^ [23], two models were made with beam elements in the structural mechanics module, under eigenfrequency analysis. The first model uses two Euler–Bernoulli beams to model a perpendicular gyroscope system that is clamped at the base with the CTBCs (4.1–4.6) applied to the tip of the Z -beam, as represented in figure 4*a*. The second model used a single upright Euler–Bernoulli beam, clamped at the base, to represent the X -beam. The Ex-CTBCs were applied to the tip of the X -beam, as indicated in figure 4*b*. Parametric sweeps were performed over the eigenfrequencies of the system for the two models, for increasing values of the gyricity Ω . The results of the parametric sweeps are overlaid on the analytical solution in figure 3 showing excellent agreement between all of the solutions.

## 6. Modal analysis of the perpendicular gyroscope

For the rest of the paper, we turn our attention to investigating the behaviour of perpendicular gyroscopic systems—whether clamped at the base or mounted on Kirchhoff plates—through studying the eigenfrequency against gyricity graphs. In this section, we analyse finite element models that were built using COMSOL Multiphysics^®^ [23], the parameters of the perpendicular gyroscope are kept the same as were given in §4, which are also listed in appendix A, table 1. The parameters of the plate have the following values, $E_{p}=70$ GPa, $\nu_{p}=0.3$ , h=0.01 m and R=1 m.

**Figure 5.**
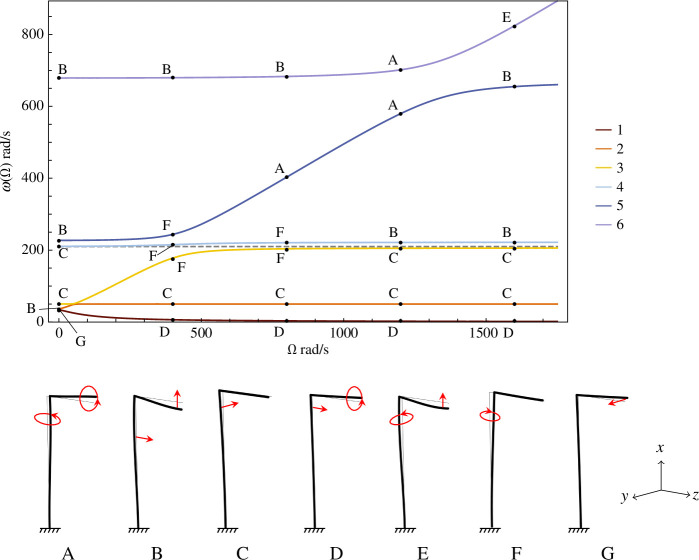
Eigenfrequencies as functions of the gyricity for a finite element model of the perpendicular gyroscope system, with a clamped base where L=0.5 m. Diagrams of the mode shapes are provided with the motions of the beam indicated by red arrows.

In figure 3, it was shown that for Ω=0 and Ω≈65 rad/s, the eigenmodes exhibit avoided crossings. The same avoided crossings of the first, second and third eigenmodes for low Ω is seen on all eigenfrequency diagrams for all systems with a perpendicular gyroscope. Figure 5 in the following section has been overlaid with the dotted line ω(Ω)=210 rad/s, showing that, while the fourth branches of each eigenfrequency diagram may initially appear flat, they do change with increasing gyricity.

### (a) Beam deformations

**Figure 6.**
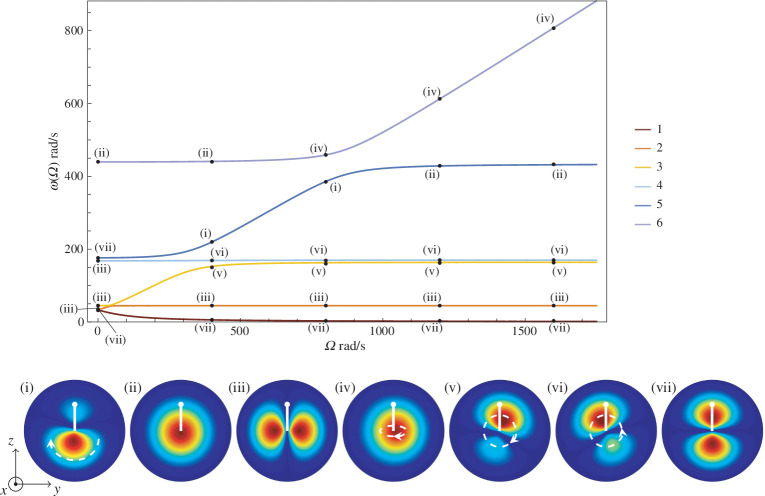
Eigenfrequencies as functions of the gyricity for the perpendicular gyroscope system with L=0.5 m mounted on a circular plate. Diagrams of plate displacements from the finite element simulations are shown below the graph. White dashed arrows indicate motion of the peak displacement of the plate.

The eigenfrequencies are plotted as functions of the gyricity for a perpendicular gyroscope that is clamped at the base in figure 5, and mounted on a circular flexural plate in figure 6, the length of the Z -beam is L=0.5 m for both. The eigenfrequencies plotted in figure 5 have precisely the same values as the analytical solutions for a clamped perpendicular gyroscopes, as shown by figure 3. Diagrams indicating the mode shapes and plate displacements are provided under each figure, and we note that both figures 5 and 6 have the same scale for ω and Ω to highlight the difference in the values of the eigenfrequencies. Nonetheless, the general shape of the eigenfrequency branches remains very similar—as is true for all of the eigenfrequency diagrams studied in this section—although the values of the eigenfrequencies become scaled depending on the conditions such as the length of the beam and shape of the plate, as will be discussed.

In figure 5, we mark the points on the eigenfrequency branches for the values of Ω=0, 400, 800, 1200 and 1600 rad/s. The mode shapes of the system are grouped into seven main types, with red arrows indicating the motion of the beams in each diagram. Mode shape A demonstrates simultaneous circling motion of both the X - and Z -beams, and is seen most on the third and fifth branches of the diagram, which experience the largest difference with increasing gyricity. Mode shapes B and C, both demonstrating flexion, are not inherently gyroscopic (as there is no circling of the beams) and are commonly seen for all different values of the gyricity, including zero gyricity.

Generally in engineering, zero eigenmodes are associated with rigid body motion. While the first eigenfrequency branch does approach zero with increasing gyricity, it does not reach zero and shows strong gyroscopic motion with circling of the tip of the Z -beam and flexural motion of the X -beam for all values of the gyricity, as shown by the mode shape diagram D. In the case of zero gyricity when the gyroscope is not spinning, there is no circling of the Z -beam and the Z -beam experiences flexion back and forth in the y -direction, as indicated by mode shape G.

Moving along the fifth eigenfrequency branch with increasing gyricity, the mode shape smoothly transitions from type B to F to A then back to B with a gradual increase then decrease in the circling of the beams. Similarly, along the sixth eigenfrequency branch, there is a smooth transition from mode shape B to A to E as Ω increases. In the regions of the avoided crossings, the mode shapes become very similar, such as for Ω=400 rad/s and Ω=1200 rad/s. We also observe mode shapes ‘jumping’ branches on the eigenfrequency diagram, for example, mode shape B can be seen jumping from the sixth eigenfrequency branch to the fifth branch after the avoided crossing. The fourth and fifth eigenfrequency branches have mode shapes C and B respectively for zero gyricity; but after the crossing, these mode shapes are seen on the fourth and third branches. Considering the fourth eigenfrequency branch alone, the perpendicular gyroscope starts by exhibiting flexural motion of mode shape C type, then exhibits increasing circling of the X -beam with increasing gyricity leading to mode shape F. As the gyricity continues to increase, the circling of the X -beam then decreases and further smooths until the mode shape only shows flexion in the two beams, indicated by mode shape B. This shows that even though the value of the eigenfrequency changes little with increasing gyricity, the mode shapes are still greatly affected.

### (b) Plate deformations

In figure 6, the eigenfrequency against gyricity graph is plotted for a perpendicular gyroscope mounted at the centre of a circular flexural plate. Compared with the clamped system shown in figure 5, the upper eigenfrequency branch and its avoided crossing becomes shifted lower in both ω and the value of gyricity at which it occurs. Examples of the displacements in the plate at different points on the branches are shown under the graph. The plates are viewed from above, with the x -axis pointing out of the page, the axis of the Z -beam points north and has been highlighted in white on each diagram. Some of the modes experience rotation around the centre of the plate, or, in the case of shape (iv), roll from one side of the plate to the other; these motions are indicated with the dashed white arrows. It is noted that when the model includes the plate, the mode shapes of the beams are largely unaffected, with two exceptions. For the sixth eigenfrequency branch in Ω∈[0,700] rad/s and the fifth branch for Ω>1000 rad/s after the avoided crossing, the beams of the perpendicular gyroscope experience compressional displacement parallel to the x -axis, following the direction of out-of-plane flexion of the plate. However, for increasing Ω , the sixth eigenmode of the perpendicular gyroscope experiences increasing flexion in the beams, returning to flexural mode shapes of type E.

**Figure 7.**
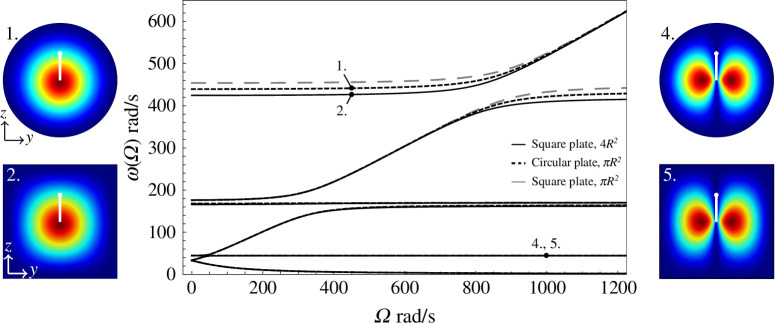
Eigenfrequencies as functions of the gyricity for different finite element models of the perpendicular gyroscope, either mounted on a square plate with area $4R^{2}$ , a circular plate with area $\pi R^{2}$ or a square plate with area $\pi R^{2}$ as indicated in the legend.

Excluding the fifth and sixth eigenfrequency branches, the shapes of the displacement in the plate generally exhibit less change moving along the eigenfrequency branches compared with the displacements of the beams. This, combined with the analysis of figure 7, leads to the conclusion that the gyroscopic motion dominates the eigenmodes, except for the sixth mode before the avoided crossing, and the fifth mode after, where the flexion of the plate dominates. The displacements of the plate do, however, experience interesting rotational motion as a consequence of the spinning gyroscope. For mode shapes (i), (v) and (vi), the plates all have a peak of maximum displacement that rotates around the base of the X -beam providing a significant difference to the usual Chladni-type patterns seen in flexural plates, which have static nodal points. The modes become increasingly similar in the region of the avoided crossing at Ω≈400 rad/s, although mode shape (vi) spins anti-clockwise whereas (i) and (v) both spin clockwise. Mode shapes (i) and (iv) become increasingly similar near the avoided crossing between the fifth and sixth eigenfrequency curves at Ω≈800 rad/s. Mode shapes (ii) and (iv) both show a single displacement peak which translates along the x -direction near the base of the X -beam, although mode shape (iv) also experiences motion side to side in the y -direction. By contrast, mode shapes (iii) and (vii) have two displacement peaks, which move anti-symmetrically to each other on either side of the base of the X -beam.

The displacements of the plate along the third, fourth and fifth eigenfrequency branches also have a marked difference between zero gyricity and when the gyricity is non-zero. For Ω=0 rad/s when the gyroscope is not spinning, these three branches start with plate displacements (iii) and (vii), which do not have any circling motion. However as the gyricity increases, the plates quickly start to show more circling motion at the base of the X -beam and the plate displacements transition to shapes (v), (vi) and (i) respectively. This demonstrates that the spinning of the gyroscope does indeed have a large effect on the motion of the plate, and could be used as a design element in platonic gratings.

### (c) Changing the shape of the Kirchhoff plate

In figure 7, the eigenfrequencies are plotted as functions of the gyricity for perpendicular gyroscopes mounted on different flexural plates: a square plate with side lengths 2R , a circular plate with radius R (as in figure 6), and a square plate with side lengths $\sqrt{\pi }R$ . The areas of the plates are therefore $4R^{2}$ , $\pi R^{2}$ and $\pi R^{2}$ , respectively. The displacements of the plate are largely unaffected when the sides of the plate lie parallel to the y - and z -directions so that the axis of the gyroscope points towards the side of the square. Comparisons of the plate displacements between circular and square plates (with side length 2R ) are shown inset in figure 7.

Excluding the sixth eigenfrequency branch before the avoided crossing, and the fifth eigenfrequency branch after the avoided crossing, the eigenfrequencies of figure 7 are in exceedingly close agreement, irrespective of the size and shape of the plate. This follows the observations from figure 6 that the lower frequency eigenmodes are dominated by the motion of the perpendicular gyroscope. The difference in the eigenfrequencies depending on the shape and area of the plate also follows the observation that when the perpendicular gyroscope is mounted on a plate, the sixth eigenmode before the avoided crossing, and the fifth eigenmode after the avoided crossing, are dominated by the motion of the plate. For large Ω , the sixth eigenfrequencies for each plate shape converge, following the observation from figure 6 that in this regime, the eigenmodes return to being dominated by the motion of the perpendicular gyroscope, and further demonstrates how the eigenmodes can jump branches on the eigenfrequency diagram in the regions of the avoided crossings.

The square plate with side lengths 2R and circular plate with radius R have different areas, so it is not unexpected that the total area of the plate has an effect on the values of the eigenfrequencies for the plate-dominated eigenmodes. However, plotting the eigenfrequencies for a square plate with side lengths $\sqrt{\pi }R$ , we see that the upper eigenfrequencies do not coincide with those from the circular plate, even though the total area is the same. We therefore conclude that both the shape and area of the plate affect the upper eigenfrequencies. This observation is in agreement with other work, studying the eigenfrequencies of membranes and plates, such as [24], which showed that the smoothness of the boundary of the membrane affected the eigenfrequencies, and membranes with sharp corners exhibited the most significant changes. It follows that we would expect to see a difference in the eigenfrequencies for a plate with a smooth circular boundary or a square plate with distinct corners.

### (d) Changing the length of the horizontal beam

**Figure 8.**
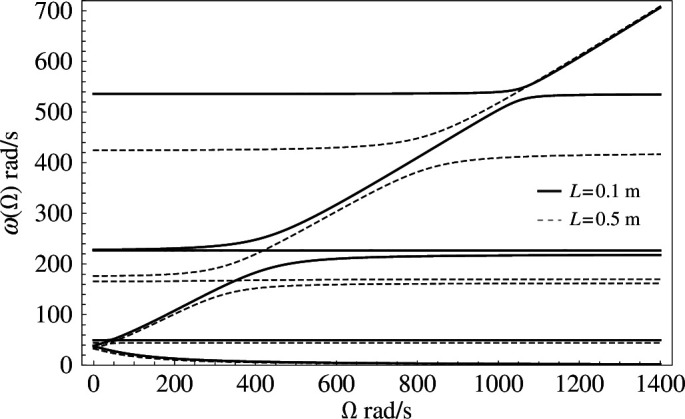
Eigenfrequencies as functions of the gyricity produced using two finite element models of perpendicular gyroscope systems on square flexural plates, with Z -beams of length L=0.5 m (dashed), and L=0.1 m (solid).

Having studied the effect of changing the shape and area of the flexural plate, we now turn our attention to how the length of the Z -beam affects the eigenfrequencies of the system. In figure 8, eigenfrequencies as functions of the gyricity are plotted for two perpendicular gyroscope systems mounted on square flexural plates with side lengths 2R , for L=0.5 m and L=0.1 m.

The two sets of solutions have many similarities, with six branches that show the same general shape and avoided crossings between all of the branches. The lowest two eigenfrequency branches for L=0.5 m and L=0.1 m are very close for all values of the gyricity, whereas the values of the upper eigenfrequencies become more significantly different. The second eigenfrequency branch of each system shows predominately type C mode shapes with flexion of the X -beam, so it would be expected that the eigenfrequency values are not largely affected by L . Both systems show type D mode shapes along the first branch, dominated by circling motion of the gyroscope, with the eigenfrequency values unaffected by the length of the Z -beam. This complements the results in §6*e*, where we compare the eigenfrequencies of parallel and perpendicular gyroscope systems. Furthermore, it has been discussed in §6*c* that for L=0.5 m the sixth eigenfrequency branch before the avoided crossing, and the fifth eigenfrequency branch after the avoided crossing, are dominated by the motion of the plate; this is also true for L=0.1 m, although the values of the upper eigenmodes are still greatly affected by the length of the Z -beam. The fact that the length of the Z -beam has such a large effect on the eigenmodes of the system, even though the plate motion dominates whether the beam is long or short, further demonstrates that the system is fully coupled. Moreover, changing the length of the Z -beam could be used as a mechanism for controlling the natural frequencies of the perpendicular gyroscope.

### (e) Similarities between parallel and perpendicular gyroscopes

In this section, we explore the similarities of the perpendicular gyroscope with a short Z -beam and the parallel gyroscope system (refer figure 1). The finite element model used to produce the eigenfrequencies of the parallel gyroscope was built in COMSOL Multiphysics^®^ [23], with the original chiral boundary conditions from [18] applied to the tip of the upright beam to model the presence of the gyroscope. All of the necessary material parameters are kept the same as the perpendicular gyroscope parameters. It is noted that the parallel gyroscope is used in this section for comparison only, a full study of the properties of parallel gyroscopes with modal analysis is provided by Nieves *et al*. in [18].

**Figure 9.**
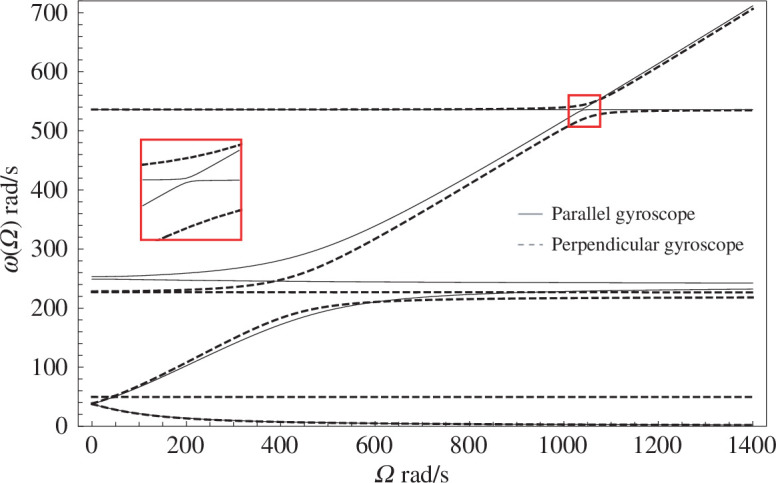
Eigenfrequencies as functions of the gyricity for the parallel gyroscope (solid lines) and the perpendicular gyroscope (dashed lines), calculated using finite element models.

In figure 9, we compare the eigenfrequencies of the perpendicular gyroscope with a Z -beam of length L=0.1 m and the parallel gyroscope, with each mounted at the centre of square plates with sides of length 2R . The proximity of the two solution sets immediately indicates multiple similarities between the two systems, although there are key differences. For both sets of solutions, all of the eigenfrequency branches do not cross—a close up of the avoided crossings at Ω≈1040 rad/s is provided inset—hence, all the eigenmodes are linearly dependent and both of the systems are fully coupled. Two systems that are said to be dynamically identical if they exhibit all of the same natural frequencies [25]. While the eigenfrequency diagram of the parallel and perpendicular gyroscope systems have some eigenmodes at the same values of ω , the eigenmode shapes of the two systems are fundamentally different.

The eigenmodes of the perpendicular gyroscope with L=0.1 m are consistent with the mode shapes depicted in figure 5 for each eigenfrequency branch; these modes are significantly different from those of the parallel gyroscope, which can only exhibit flexion and circling of the X -beam. It is also noted that the parallel gyroscope system only has five eigenfrequencies, compared with the six eigenfrequencies of the perpendicular gyroscope. This is owing to the lack of rotational symmetry about the x -axis for the perpendicular gyroscope system, which necessitates the inclusion of the torsional boundary condition (4.6). As discussed above for the perpendicular gyroscope, the sixth eigenmode before the avoided crossing, and the fifth mode after, are dominated by motion of the plate. The same can be said for the parallel gyroscope with the fifth eigenmode before the avoided crossing, and fourth after, where the solution sets for each system are almost identical. The first, lowest, eigenfrequency branch of figure 9 also has very close values for both the parallel and perpendicular systems. For a parallel gyroscope, this mode is dominated by circling motion of the X -beam about the x -axis. In contrast, the mode shape for the perpendicular gyroscope system shows circling of the Z -beam with minimal movement of the X -beam for all values of the gyricity, shown by mode shape D from (figure 5).

While it is untrue to say that the perpendicular gyroscope and parallel gyroscope could ever be equivalent, and the differences between the eigenfrequency diagrams cannot be overlooked, this analysis shows that the Z -beam needs for be sufficiently long for the flexion of the beam to affect the values of the eigenfrequencies. Compared with figure 9, there is very little change to the eigenfrequencies of the perpendicular gyroscope when 0<L≤0.1 . When L becomes very small, the perpendicular gyroscope system visually appears more similar to the parallel gyroscope system, excluding the direction of the axis of the gyroscope, however the asymmetry of the system is always non-negligible.

## 7. Concluding remarks

This paper has presented an analytical model of a perpendicular gyroscope system composed of two perpendicular beams, a gyroscope and a flexural plate. Finite element simulations were used to complement the analytical results and study the eigenmodes of the system. The coupling of flexural and torsional rotations was discussed in detail, with formulae describing the translation of flexural, compressional and torsional forces and moments across the junction between the beams provided in §3. A set of extended chiral-torsion boundary conditions were derived to simulate the presence of a flexural beam and gyroscope combination, allowing for the entire perpendicular gyroscope system to be modelled using only a single upright beam. Modal analysis in §6 revealed a fully coupled system that is sensitive to material parameters such as the beam lengths, plate shapes and rate of spin of the gyroscope; this coupling means that each mode cannot be considered independently when analysing the structure.

In the final section of this work, comparisons were made between the perpendicular gyroscope and a single upright beam with an attached spinner. The two systems were shown to have many similarities but also key differences. The perpendicular gyroscope offers more mechanisms for controlling the natural frequencies and dynamic behaviour of the system; for example, by changing the length and/or angle of the horizontal beam. The presence of the secondary beam also means that the perpendicular gyroscope exhibits significantly different mode shapes to the parallel gyroscope. This arises from the coupling of compressional, flexural and torsional modes, which are all decoupled for parallel gyroscopes.

The work in this paper establishes a mechanism for studying periodic arrays of perpendicular gyroscopes; for example by using the perpendicular gyroscope on a square plate as a repeating unit cell. Arrays of gyroscopes and beams have been studied in other works such as [2,3,7,8] with interesting applications for designing topological insulators capable of isolating vibrations in chosen regions. This work has particular applications to systems of spinning rotors where the axis of rotation is perpendicular or near perpendicular to the axis at the base, such as in wind turbines. Furthermore, analysing the deformations caused by the base of the perpendicular gyroscope could, in future, provide insights on the vibrations of the sea bed for off-shore wind farms, which are known to affect the surrounding marine ecosystems [26,27].

## Figures and Tables

**Figure 10.**
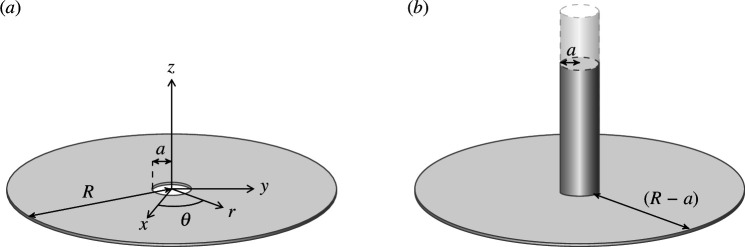
(*a*) A circular plate with outer radius R , which has an inclusion of radius a . (*b*) The end of a cylindrical beam sits inside the inclusion of the plate. It is noted that the ratio of a to R has been exaggerated in the figure for the purpose of illustration.

## Data Availability

This article has no additional data.

## Appendix A. Table of parameters

See table 1.

**Table 1.** A summary of the parameters associated with the perpendicular gyroscope system. The values of L and Ω have been omitted as the effect of changing these parameters is investigated in this work.

parameter	description	value
H	height of the X -beam	1 m
L	length of the Z -beam	—
$a_{x}$	radius of the X -beam	0.02 m
$a_{z}$	radius of the Z -beam	0.02 m
E	Young’s modulus of the Z -beam	70 GPa
E	Young’s modulus of the X -beam	70 GPa
$I_{x}$	second moment of area in the x -direction for the Z -beam	$4\pi \times 10^{-8}$ m ${,}^{4}$
$I_{y}$	second moment of area in the y -direction for the Z -beam	$4\pi \times 10^{-8}$ m ${,}^{4}$
$I_{y}$	second moment of area in the y -direction for the X -beam	$4\pi \times 10^{-8}$ m ${,}^{4}$
$I_{z}$	second moment of area in the z -direction for the X -beam	$4\pi \times 10^{-8}$ m ${,}^{4}$
$A_{z}$	cross-sectional area for the Z -beam	$4\pi \times 10^{-4}$ m ${,}^{2}$
$A_{x}$	cross-sectional area for the X -beam	$4\pi \times 10^{-4}$ m ${,}^{2}$
J	torsion constant in the z -direction for the Z -beam	$8\pi \times 10^{-8}$ m ${,}^{4}$
J	torsion constant in the x -direction for the X -beam	$8\pi \times 10^{-8}$ m ${,}^{4}$
G	shear modulus for the Z -beam	26 GPa
G	shear modulus for the X -beam	26 GPa
R	radius of the circular plate	1 m
h	thickness of the plate	0.01 m
$E_{p}$	Young’s modulus of the plate	70 GPa
$\nu_{p}$	Poisson’s ratio for the plate	0.3
D	flexural rigidity of the plate	6410 Pa m ${,}^{3}$
m	mass of the gyroscopic spinner	1 kg
$I_{0}$	moment of inertia of the gyroscope about the x - and y -axes	4 kg m ${,}^{2}$
$I_{1}$	moment of inertia of the gyroscope about the z -axis	2 kg m ${,}^{2}$
Ω	gyricity associated with the gyroscope	—

## Appendix B. Connecting beams to plates

In §2 of this work, we use a set of boundary conditions—in the form of forces and moments—to describe the connection between a flexural plate and an upright flexural beam. The conditions were first derived in the paper [1] to describe the connection between a beam and a plate with non-zero density. Taking the static limit such that ω→0 (or equivalently so that the density goes to zero) and balancing the forces and moments with those at the end of a flexural beam led to the *logarithmic boundary conditions*. The original boundary conditions were rotated and translated to be suitable for the perpendicular gyroscope in §2.

Here, we provide an alternative derivation of the logarithmic boundary conditions starting with a massless plate so that the Kirchhoff–Love plate equation immediately simplifies to the homogeneous biharmonic equation. We first derive the forces and moments caused by displacements at the edge of a circular inclusion in a thin flexural plate, then balance the forces and moments with those at the end of a flexural beam that sits inside the inclusion. Figure 10 illustrates a plate with an inclusion, and the plate–beam connection. It is noted that this approach to derive the logarithmic boundary conditions is suitable for beams with circular cross-sections. For beams with non-circular or polygonal cross-sections, the shape of the inclusion in the plate would need to be altered to coincide with the shape of the beam cross-section. In turn, new boundary conditions would be required to account for the different forces and moments that occur at the edge of the inclusion when the system does not have radial symmetry.

 To start, we consider a circular flexural plate in the xy -plane, centred at the origin, with radius R and clamped at the boundary. The centre of the plate has a circular inclusion with radius a<R , as shown in figure 10*a*. In polar coordinates (r,θ) , the equation governing the displacement of the plate in the z -direction is

(B 1)
$$D\Delta^{2}w_{p}(r,\theta )=0\;\;\;\;\text{on}\;\;\;\;\Sigma =\{(r,\theta )\;:\;a\leq r\leq R\},$$

with boundary conditions $w_{p}(R,\theta )=0$ and $\partial_{r}w_{p}(R,\theta )=0.$ For massless plates such that $\rho_{p}=0$ , the homogeneous biharmonic (B 1), can be solved by the Michell solution [28],

(B 2)
$$\begin{array}{c} w_{p}\left(r,\theta \right)=A_{0}r^{2}+B_{0}r^{2}ln\left(r\right)+C_{0}ln\left(r\right)+\left(I_{0}r^{2}+I_{1}r^{2}ln\left(r\right)+I_{2}ln\left(r\right)+I_{3}\right)\theta \\ \;\;\;\;+\left(A_{1}r+B_{1}r^{-1}+{\overset{˜}{B}}_{1}r\theta +C_{1}r^{3}+D_{1}rln\left(r\right)\right)cos\left(\theta \right)\\ \;\;\;\;+\left(E_{1}r+F_{1}r^{-1}+{\overset{˜}{F}}_{1}r\theta +G_{1}r^{3}+H_{1}rln\left(r\right)\right)sin\left(\theta \right)\\ \;\;\;\;+\sum_{n=2}^{\infty }\left(A_{n}r^{n}+B_{n}r^{-n}+C_{n}r^{n+2}+D_{n}r^{2-n}\right)cos\left(n\theta \right)\\ \;\;\;\;+\sum_{n=2}^{\infty }\left(E_{n}r^{n}+F_{n}r^{-n}+G_{n}r^{n+2}+H_{n}r^{2-n}\right)sin\left(n\theta \right). \end{array}$$

The inclusion is subject to a rotation $\alpha_{y}$ about the y -axis, causing displacement of the plate in the z -direction. This gives the boundary conditons for the inclusion, $w_{p}(a,\theta )=-\alpha_{y}a\cos(\theta )$ and $\partial_{r}w_{p}(a,\theta )=-\alpha_{y}\cos(\theta ).$ From this, we know that the solution for $w_{p}$ must be of the form $w_{p}(r,\theta )=f(r)\cos(\theta )$ and using the Michell solution gives

(B 3)
$$w_{p}(r,\theta )=\left(A_{1}r+B_{1}r^{-1}+C_{1}r^{3}+D_{1}r\ln(r)\right)\cos(\theta ).$$

Combining the boundary conditions at the inclusion and edge of the plate with (B 3), gives the following relations,

$$\begin{array}{ll} -\alpha_{y}acos\left(\theta \right) & =\left(A_{1}a+B_{1}a^{-1}+C_{1}a^{3}+D_{1}aln\left(a\right)\right)cos\left(\theta \right)\\ -\alpha_{y}cos\left(\theta \right) & =\left(A_{1}-B_{1}a^{-2}+3C_{1}a^{2}+D_{1}\left(1+ln\left(a\right)\right)\right)cos\left(\theta \right)\\ \;\;\;\;\;0 & =\left(A_{1}R+B_{1}R^{-1}+C_{1}R^{3}+D_{1}Rln\left(R\right)\right)cos\left(\theta \right)\\ \;\;\;\;\;0 & =\left(A_{1}-B_{1}R^{-2}+3C_{1}R^{2}+D_{1}\left(1+ln\left(R\right)\right)\right)cos\left(\theta \right). \end{array}$$

Solving the above system of simultaneous equations for $A_{1}$ , $B_{1}$ , $C_{1}$ and $D_{1}$ leads to the solution

(B 4)
$$w_{p}\left(r,\theta \right)=\left[\left(r^{2}-1\right)\left(r^{2}+\frac{a^{2}}{R^{2}}\right)-2r^{2}\left(1+\frac{a^{2}}{R^{2}}\right)ln\left(r\right)\right]\frac{cos\left(\theta \right)}{r\gamma },$$

where

$$\gamma =1-\frac{a^{2}}{R^{2}}+\left(1+\frac{a^{2}}{R^{2}}\right)ln\left(\frac{a}{R}\right).$$

The radial moment and shear force induced by flexural motion in the plate are [29,30],

$$M_{r}=D\left[\frac{\partial^{2}w_{p}}{\partial r^{2}}+\nu_{p}\left(\frac{1}{r}\frac{\partial w_{p}}{\partial r}+\frac{1}{r^{2}}\frac{\partial^{2}w_{p}}{\partial \theta^{2}}\right)\right],\;\;\;\;V_{r}=D\frac{\partial }{\partial r}\left[\frac{\partial^{2}w_{p}}{\partial r^{2}}+\frac{1}{r}\frac{\partial w_{p}}{\partial r}+\frac{1}{r^{2}}\frac{\partial^{2}w_{p}}{\partial \theta^{2}}\right].$$

Using (B4), the moment about the y -axis is given by [31],

(B 5)
$$M_{y}=\int_{0}^{2\pi }\left(M_{r}-aV_{r}\right){|}_{r=a}acos\left(\theta \right)\;d\theta \;\;\;\;=4\pi \frac{\alpha_{y}D}{\gamma }\left(1+\frac{a^{2}}{R^{2}}\right).$$

It is noted that for the angle $\alpha_{y}$ about the y -axis, the induced moment about the x -axis is given by

$$M_{x}={\int_{0}^{2\pi }\left(-M_{r}+aV_{r}\right)|}_{r=a}a\sin(\theta )\;d\theta$$

and so, for angles about the y -axis, $M_{x}=0$ . Considering an angle of rotation of the inclusion $\alpha_{x}$ about the x -axis, and repeating the above steps for calculating the resulting moments about the x - and y -axes leads to $M_{y}=0$ and

(B 6)
$$M_{x}=4\pi \frac{\alpha_{x}D}{\gamma }\left(1+\frac{a^{2}}{R^{2}}\right).$$

 However, considering a translational displacement of magnitude Z along the z -axis at the boundary of the inclusion r=a , the displacement of the plate has no dependence on θ and we must use a different approach to above. In the case when the system has no angular dependence, the most convenient solution of the biharmonic equation is the axisymmetric solution [30],

(B 7)
$$w_{p}(r)=\frac{1}{2}C_{2}r^{2}-\frac{1}{4}C_{3}r^{2}+C_{4}+\left(C_{1}+\frac{1}{2}C_{3}r^{2}\right)\ln(r).$$

For the displacement Z , the boundary conditions at the inclusion are $w_{p}(a)=Z$ and $\partial_{r}w_{p}(a)=0$ . In the axisymmetric case, the shear force in the z -direction is given by [31]

$$V_{z}={\int_{0}^{2\pi }V_{r}|}_{r=a}a\;d\theta =2\pi D\left[\frac{C_{1}}{a^{2}}+C_{2}+\left(\ln(a)+2\right)C_{3}\right].$$

Using the boundary conditions for the inclusion and the edge of the plate allows us to find the coefficients $C_{i}$ from (B 7), and we arrive at the expression for the shear force

(B 8)
$$V_{z}=16\pi \frac{D}{\eta }\left(a^{2}-R^{2}\right)\;\;\;\;\text{where}\;\;\;\;\eta ={\left(a^{2}-R^{2}\right)}^{2}-4a^{2}R^{2}\ln^{2}(a/R).$$

It is noted that the expressions for $M_{y}$ , $M_{x}$ and $V_{z}$ from (B 5), (B 6) and (B 8) agree exactly with the derived results for the forces and moments at the inclusion from [1] for the static limit, however without the need to consider the dynamic Green’s function of the plate.

 As was done in [1], we now consider the case when a cylindrical beam parallel to the z -axis, with cross-sectional radius a , is placed inside the inclusion, as illustrated in figure 10*b*. The moments from (B 5) and (B 6) then correspond to the moments applied to the end of the beam by the presence of the plate, and equation (B 8) corresponds to the compressional force applied to the beam by the plate. Assuming the junction of the beam and plate is located at the origin, angles $\alpha_{y}$ and $\alpha_{x}$ coincide with the flexural rotation angles $\partial_{z}u(z)$ and $\partial_{z}v(z)$ at the z=0 end of the beam in the x - and y -directions, respectively. Similarly, a displacement Z of the inclusion would produce a displacement w(z) in the beam at z=0 . Balancing the moments at the inclusion about the x - and y -axes, with the flexural bending moments in the beam leads to the following relations

$$EI_{x}\frac{\partial^{2}}{\partial z^{2}}u\left(0\right)=-\frac{4\pi D\left(1+{\left(a/R\right)}^{2}\right)}{1-{\left(a/R\right)}^{2}+\left(1+{\left(a/R\right)}^{2}\right)\text{ln}\left(a/R\right)}\partial_{z}u\left(0\right),$$

$$EI_{y}\frac{\partial^{2}}{\partial z^{2}}v\left(0\right)=-\frac{4\pi D\left(1+{\left(a/R\right)}^{2}\right)}{1-{\left(a/R\right)}^{2}+\left(1+{\left(a/R\right)}^{2}\right)ln\left(a/R\right)}\partial_{z}v\left(0\right).$$

Balancing the compressional force at the end of the beam with the force from the plate gives

$$EA\frac{\partial }{\partial z}w\left(0\right)=\frac{16\pi D\left(1-{\left(a/R\right)}^{2}\right)}{R^{2}\left[{\left(1-{\left(a/R\right)}^{2}\right)}^{2}-4{\left(a/R\right)}^{2}ln^{2}\left(a/R\right)\right]}w\left(0\right).$$

In the limit that a/R≪1 , the above relations, to the leading order, are

(B 9)
$$EI_{x}\frac{\partial^{2}}{\partial z^{2}}u\left(0\right)=-\frac{4\pi D}{\text{ln}\left(a/R\right)}\partial_{z}u\left(0\right),$$

(B 10)
$$EI_{y}\frac{\partial^{2}}{\partial z^{2}}v\left(0\right)=-\frac{4\pi D}{\text{ln}\left(a/R\right)}\partial_{z}v\left(0\right),$$

(B 11)
$$EA\frac{\partial^{2}}{\partial z^{2}}w\left(0\right)=\frac{16\pi D}{R^{2}}w\left(0\right).$$

## Appendix C. Relationships between the coefficients of the displacements

In §3, kinematic and dynamic condition are provided to describe the coupling of displacements, rotations, forces and moments across the perpendicular junction between the X -beam and Z -beam. The conditions are essential for solving the eigenvalue problem from §4 and 5. The conditions (2.1) lead to the following relations between the coefficients of the X -beam and the Z -beam,

(C 1)
$$\begin{array}{ll} {\overset{˜}{u}}_{1}H+U_{p} & =u_{0}\\ {\overset{˜}{v}}_{3}H^{3}+{\overset{˜}{v}}_{2}H^{2}+\varphi_{z}H & =v_{0}\\ {\overset{˜}{w}}_{3}H^{3}+{\overset{˜}{w}}_{2}H^{2}+\varphi_{y}H & =w_{0} \end{array}\;\;\;\;\;\;\begin{array}{ll} {\overset{˜}{\tau }}_{1}H & =v_{1}\\ 3{\overset{˜}{w}}_{3}H^{2}+2{\overset{˜}{w}}_{2}H+\varphi_{y} & =u_{1}\\ 3{\overset{˜}{v}}_{3}H^{2}+2{\overset{˜}{v}}_{2}H+\varphi_{z} & =\tau_{0}. \end{array}$$

The balance of force and moments at the junction (3.2)–(3.4) gives the following relations between the higher order coefficients of the Z -beam displacements and the coefficients from the X -beam displacements,

(C 2)
$$\begin{array}{ll} -6EI_{x}u_{3} & =EA_{x}{\overset{˜}{u}}_{1}\\ 6EI_{y}v_{3} & =6{EI}_{y}{\overset{˜}{v}}_{3}\\ EA_{z}w_{1} & =-6{EI}_{z}{\overset{˜}{w}}_{3} \end{array}\;\;\;\;\;\;\begin{array}{ll} 2EI_{y}v_{2} & =JG{\overset{˜}{\tau }}_{1}\\ 2EI_{x}u_{2} & =6{EI}_{z}{\overset{˜}{w}}_{3}H+2{EI}_{z}{\overset{˜}{w}}_{2}\\ JG\tau_{1} & =6{EI}_{y}{\overset{˜}{v}}_{3}H+2{EI}_{y}{\overset{˜}{v}}_{2}. \end{array}$$

Combining (B 1) and (B 2) with the relations between the Z -beam coefficients and the angles at the junctions (4.8), we can write the higher order coefficients of the Z -beam displacements exclusively in terms of the displacements at the junction and the displacements of the plate as follows:

$$\begin{array}{ll} u_{3} & =-\frac{{EA}_{x}}{6EI_{x}H}\left(u_{0}-U_{p}\right)\\ v_{3} & =\frac{{EI}_{y}}{EI_{y}H^{3}}\left(-\theta_{z}H-2v_{0}+\varphi_{z}H\right)\\ w_{1} & =-\frac{6{EI}_{z}}{EA_{z}H^{3}}\left(\theta_{y}H-2w_{0}+\varphi_{y}H\right) \end{array}\;\;\;\;\;\;\begin{array}{ll} v_{2} & =-\frac{JG}{2EI_{y}}\frac{\theta_{x}}{H}\\ u_{2} & =\frac{{EI}_{z}}{2EI_{x}H^{2}}\left(4\theta_{y}H-6w_{0}+2\varphi_{y}H\right)\\ \tau_{1} & =\frac{{EI}_{y}}{JGH^{2}}\left(-4\theta_{z}H-6v_{0}+2\varphi_{z}H\right). \end{array}$$
